# Supplementary material for: Transcription profiles reveal sugar and hormone signaling pathways mediating tree branch architecture in apple (Malus domestica Borkh.) grafted on different rootstocks
Source: PLoS One. 2020 Jul 24;15(7):e0236530. doi: 10.1371/journal.pone.0236530 (PMC7380599; doi:10.1371/journal.pone.0236530)
Supplement: S1 Table — (DOCX) [file pone.0236530.s003.docx]

**Table S1 List of primers used for real-time PCR.**

| **Genes** | **Forward (5’ to 3’)** | **Reverse (5’ to 3’)** |
| --- | --- | --- |
| *SUS4* | CAGCTTTGAAGCGATCTG | CGACTGAGGATGTAACCA |
| *GolS1-like* | CAGAAGTTCCAGCTGATG | CACGTCCGGCAATATCGC |
| *SPS4* | CGACTGGTTAAACGGTTA | CTGTGTTCTCGGCTATTC |
| *SS5-like* | CGTCAAACGCTCCGATTC | TAACAACAGCTTCCTGAG |
| *AGLA* | GATCGTCTAAGAATTCGA | GTATTGGTCCTTGAAGAC |
| *GIL* | GCTACTACTTGTTGTAGT | GCTGTTGAGGATGGTGAC |
| *S6PDHL* | CACTCAACAGTGGGTTCA | CAAATGATTCTGAGAACG |
| *β-GAL* | CACAGAAGAGCAGGAAAC | CCTCAGTTGAACGGCTGA |
| *UGTL* | GCCATATCAAGGCCATGC | ACAGAGTCAGCGAGCAAG |
| *ABA8OX4* | CACTTCCGCCGGGTTCCT | AAGTTGGCTTGAACAGAT |
| *RGL2-2* | GTTCAACTCGGTCTTCGA | CACCCTAGGGATGCCATG |
| *PIN3* | GAAGATGTTTACAAGGTG | ATGAAGTCGGCGCCGATG |
| *ERF034-LIKE* | CAATTCATTGACGGATCA | GCTCGCGGATTTCCGACA |
| *ARF7-LIKE* | GACAGCTCACGAGTCACG | CTCCATCCAGTTGTGAGC |
| *COL4* | CATATTGGGGGACTACTA | CTAGATGAGGAGATCCTG |
| *STL* | AGGAGCTAGCTGTAGAGT | GCCCAAATATCCACTGCA |
| *ERF105* | ATGGATAGCTTCATCACT | CGAATTGGGTGTCTCGAT |
| *MYC2* | GTTCATCAGATCGGAGGT | CAGACGACGCCGTCGTTG |
