# Supplementary material for: Transcription profiles reveal sugar and hormone signaling pathways mediating tree branch architecture in apple (Malus domestica Borkh.) grafted on different rootstocks
Source: PLoS One. 2020 Jul 24;15(7):e0236530. doi: 10.1371/journal.pone.0236530 (PMC7380599; doi:10.1371/journal.pone.0236530)
Supplement: S2 Table — (DOCX) [file pone.0236530.s004.docx]

**Table S2 Summary of the sequencing data in each sample.**

| Sample | Total Reads | Mapped Reads | Mapped Ratio % | Uniq Mapped Reads | Uniq Mapped Ratio % | Base Number | GC% | Q30% |
| --- | --- | --- | --- | --- | --- | --- | --- | --- |
| VR-1 | 49,306,876 | 44,587,788 | 90.43 | 39,205,595 | 79.51 | 7,396,031,400 | 47.42 | 93.55 |
| VR-2 | 44,627,974 | 40,715,452 | 91.23 | 35,773,091 | 80.16 | 6,694,196,100 | 47.17 | 92.88 |
| VR-3 | 43,321,986 | 39,658,072 | 91.54 | 35,106,022 | 81.04 | 6,498,297,900 | 47.92 | 93.38 |
| DIR-1 | 43,456,110 | 39,346,026 | 90.54 | 34,241,436 | 78.80 | 6,518,416,500 | 47.20 | 92.09 |
| DIR-2 | 44,489,016 | 40,419,066 | 90.85 | 35,257,566 | 79.25 | 6,673,352,400 | 47.40 | 93.28 |
| DIR-3 | 41,293,076 | 37,128,060 | 89.91 | 32,285,268 | 78.19 | 6,193,961,400 | 47.43 | 93.00 |
| DSR-1 | 44,139,808 | 39,678,750 | 89.89 | 34,659,367 | 78.52 | 6,620,971,200 | 47.33 | 92.49 |
| DSR-2 | 43,172,020 | 38,896,478 | 90.10 | 33,808,760 | 78.31 | 6,475,803,000 | 46.87 | 92.27 |
| DSR-3 | 41,127,306 | 37,257,072 | 90.59 | 32,933,443 | 80.08 | 6,169,095,900 | 47.74 | 93.46 |

Note: Samples VR-1, -2, and -3 were leaves of apple trees on vigorous rootstock; Samples DIR-1, -2, and -3 were leaves of apple trees on dwarfing interstock; Samples DSR-1, -2, and -3 were leaves of apple trees on dwarfing self-rootstock; Total reads: the number of clean reads; Mapped Reads: the number of clean reads located in the reference genome; Mapped Ratio %: percentage of clean reads located in the reference genome in all clean reads; Uniq Mapped Reads: number of reads compared to the unique position of the reference genome; Uniq Mapped Ratio %: percentage of reads compared to the unique position of the reference genome; Base Number: the number of filtered base; GC%: the percentage of G and C types of bases in the total base; Q30(%): the percentage of bases with a mass value greater than or equal to 30 in the total base number.
