# Supplementary material for: Transcription profiles reveal sugar and hormone signaling pathways mediating tree branch architecture in apple (Malus domestica Borkh.) grafted on different rootstocks
Source: PLoS One. 2020 Jul 24;15(7):e0236530. doi: 10.1371/journal.pone.0236530 (PMC7380599; doi:10.1371/journal.pone.0236530)
Supplement: S3 Table — (DOCX) [file pone.0236530.s005.docx]

| Group Set | All DEG | Up-regulated | Down-regulated |
| --- | --- | --- | --- |
| VR *vs* DIR | 2510 | 1394 | 1116 |
| VR *vs* DSR | 1651 | 1164 | 487 |
| DIR *vs* DSR | 1153 | 908 | 245 |

**Table S3 Statistics of the DEG numbers among different groups.**
