# Supplementary material for: Transcription profiles reveal sugar and hormone signaling pathways mediating tree branch architecture in apple (Malus domestica Borkh.) grafted on different rootstocks
Source: PLoS One. 2020 Jul 24;15(7):e0236530. doi: 10.1371/journal.pone.0236530 (PMC7380599; doi:10.1371/journal.pone.0236530)
Supplement: S4 Table — (DOCX) [file pone.0236530.s006.docx]

**Table S4 Information of selected sugar-related genes in leaves of apple trees on different rootstocks.**

| **Gene ID** | **FPKM** | | | **Annotation** |
| --- | --- | --- | --- | --- |
|  | **VR** | **DIR** | **DSR** |  |
| **Cluster 1** |  |  |  |  |
| MD06G1237200 | 1.893231 | 2.429728 | 1.672984 | sucrose synthase 4 (SUS4) |
| MD09G1280200 | 1.635829 | 2.370363 | 1.443365 | sucrose synthase 5 (SUS5) |
| MD14G1244000 | 1.787287 | 2.258898 | 1.351292 | sucrose synthase 6-like (SUSL-6) |
| MD01G1169500 | 1.746574 | 3.581269 | 3.552803 | uncharacterized aarF domain-containing protein kinase (FITC) |
| MD02G1273700 | 1.224315 | 2.625314 | 3.486551 | rust resistance kinase Lr10-like (RRKL10) |
| MD01G1182600 | 0.043329 | 4.118063 | 4.062482 | peroxisomal membrane protein 11B-like (PEX11B) |
| apple_newGene_2022 | 0 | 4.497999 | 4.355282 | RGL2-2 |
| MD03G1091100 | 0.779296 | 3.717182 | 3.01604 | (R)-mandelonitrile lyase 2-like (RMDL2) |
| MD16G1095000 | 1.212976 | 3.264136 | 2.809111 | galactinol synthase 1-like (GolS1-like) |
| MD06G1023200 | 0.129653 | 2.891756 | 2.660844 | thiamine pyrophosphokinase 1-like (TPS1-LIKE) |
| MD07G1279500 | 0.126548 | 2.945161 | 2.523278 | uncharacterized protein PAM68-like |
| apple_newGene_2510 | 0.423161 | 2.960459 | 2.307279 | decapping and exoribonuclease protein-like (DXO) |
| MD11G1004800 | 0.108166 | 2.899812 | 2.166761 | alcohol dehydrogenase 1-like (ADH1-LIKE) |
| **Cluster 2** |  |  |  |  |
| MD12G1186000 | 2.668426 | 3.260548 | 4.983988 | serine/threonine-protein kinase WNK5 (PK5) |
| MD06G1027300 | 2.194413 | 3.006459 | 4.639085 | xyloglucan endotransglucosylase/hydrolase protein 30 (XTH30) |
| MD13G1177500 | 1.905379 | 2.466797 | 4.590406 | phosphate dikinase (PDK) |
| MD05G1013400 | 1.345527 | 1.869808 | 3.791419 | alcohol dehydrogenase 1 (ADH1) |
| MD10G1240300 | 3.018135 | 1.68228 | 2.700691 | alpha-glucosidase (AGLA) |
| MD11G1079800 | 4.14496 | 1.877802 | 3.215029 | 50S ribosomal protein L4 (RPLD) |
| MD00G1105700 | 7.350986 | 2.866266 | 5.209122 | beta-glucosidase 12-like (BGLU12-LIKE) |
| MD11G1146700 | 4.042242 | 3.18619 | 2.80472 | xyloglucan endotransglucosylase/hydrolase 31-like (XTH31-LIKE) |
| apple_newGene_171 | 3.405946 | 2.424607 | 2.699846 | ATP-dependent zinc metalloprotease (FtsH-like) |
| apple_newGene_2513 | 3.639278 | 2.425338 | 2.665737 | GDT1-like protein 4 (GDTL-4) |
| MD00G1054500 | 3.712368 | 2.429017 | 2.886477 | 2-hydroxyacyl-CoA lyase (HACL) |
| MD13G1106200 | 4.15393 | 2.57271 | 2.604517 | pectate lyase 5 (PEL5) |
| MD15G1021100 | 3.870891 | 2.390648 | 2.206862 | beta-galactosidase 5 (β-GAL5) |
| MD10G1169100 | 3.603693 | 2.862062 | 2.120878 | pectate lyase 18 (PEL18) |
| MD12G1089500 | 3.537892 | 5.439819 | 6.32977 | glucan endo-1,3-beta-glucosidase-like (GIL) |
| MD13G1093700 | 4.542904 | 5.338245 | 7.243646 | galactinol synthase 1-like (GolS1-like) |
| MD15G1020800 | 4.764548 | 5.023607 | 5.968511 | lon protease 2-like (IL2) |
| MD16G1014000 | 5.153199 | 5.299494 | 6.427606 | xyloglucan endotransglucosylase/hydrolase protein 28 (XTH28) |
| MD16G1179400 | 4.124435 | 4.369306 | 5.409596 | phosphate dikinase 1 (PDK1) |
| MD15G1099500 | 4.142732 | 4.613062 | 5.690245 | two-on-two hemoglobin-3 (GLB3) |
| MD10G1012400 | 5.040259 | 5.716082 | 6.057321 | psbP domain-containing protein 4 (PSPB4) |
| MD08G1023400 | 5.591315 | 6.029874 | 6.819628 | lon protease 2 (IP2) |
| MD10G1249200 | 2.154017 | 2.529844 | 2.912061 | wall-associated receptor kinase 2-like (WAK2L) |
| MD11G1093500 | 5.6977 | 6.39812 | 7.048842 | tropinone reductase (TR) |
| MD05G1006400 | 3.081571 | 3.803091 | 4.31747 | sucrose-phosphate synthase 4 (SPS4) |
| MD16G1022000 | 4.408913 | 5.565255 | 6.292681 | pectinesterase-like (PECSL) |
| MD02G1192600 | 6.16869 | 7.683818 | 8.043062 | xyloglucan endotransglucosylase/hydrolase protein 6 (XTH6) |
| MD16G1022100 | 5.308908 | 6.298191 | 6.979489 | pectinesterase-like (PECSL) |
| MD05G1054800 | 3.633892 | 4.937743 | 5.450609 | NADP-dependent D-sorbitol-6-phosphate dehydrogenase-like (S6PDH) |
| MD02G1247300 | 1.921253 | 2.752893 | 2.823339 | rust resistance kinase Lr10-like (RRKL10) |
| MD08G1107600 | 1.988995 | 2.895085 | 2.962892 | wall-associated receptor kinase-like 16 (WAK16) |
| MD02G1264600 | 2.411863 | 3.988865 | 4.10498 | NADP-dependent D-sorbitol-6-phosphate dehydrogenase-like (S6PDHL) |
| MD04G1020100 | 6.490901 | 7.242587 | 6.085136 | xyloglucan endotransglucosylase/hydrolase 2 (XTH2) |
| MD09G1145500 | 3.047211 | 4.095521 | 3.946097 | wall-associated receptor kinase-like 22 (WAK22) |
| MD11G1093900 | 5.472124 | 7.284517 | 6.164935 | tropinone reductase homolog (TR) |
| MD08G1057400 | 3.626125 | 5.273999 | 3.867316 | chorismate mutase 2-like (CML2) |
| MD13G1064400 | 6.761243 | 6.424373 | 5.746995 | UDP-glucose 6-dehydrogenase 5-like (UGDH5-LIKE) |
| MD16G1106400 | 6.572257 | 5.635892 | 5.478151 | pectate lyase 5 (PL5) |
| apple_newGene_1502 | 5.659885 | 4.633265 | 4.968282 | cytochrome b-c1 complex subunit 6-like (CCS6-LIKE) |
| MD13G1231000 | 4.938989 | 4.205349 | 3.747952 | pectate lyase 8 (PL8) |
| MD17G1286900 | 2.892689 | 3.74402 | 2.666527 | sucrose synthase 5-like (SS5-LIKE) |
| MD15G1040000 | 3.139555 | 4.342424 | 2.599285 | chorismate mutase 2 (CM2) |
| MD06G1200200 | 3.463457 | 3.630168 | 2.684172 | UDP-glucose 6-dehydrogenase 3 (UGD3) |
| MD04G1198000 | 3.683764 | 3.995201 | 3.058004 | pectinesterase/pectinesterase inhibitor 22 (PME22) |
| MD10G1013900 | 1.759752 | 2.60877 | 2.220996 | alcohol dehydrogenase (ADH1) |
| **Cluster 3** |  |  |  |  |
| MD15G1088400 | 2.009446 | 0.761196 | 2.283816 | wall-associated receptor kinase-like 16 (WAK16L) |
| apple_newGene_1867 | 2.68336 | 0 | 2.46893 | peptidyl-prolyl cis-trans isomerase (CYP19-3-like) |
| MD05G1288600 | 2.552832 | 0.033963 | 1.788263 | beta-xylosidase/alpha-L-arabinofuranosidase 1-like (XYL1) |
| MD10G1209200 | 3.682437 | 0.11375 | 3.169154 | ATP-dependent zinc metalloprotease (FTSH12) |
| apple_newGene_2508 | 3.591223 | 0.040538 | 3.15486 | vesicle-fusing ATPase-like (VFAL) |
| MD10G1083900 | 3.254008 | 0.835501 | 2.035807 | beta-galactosidase (β-GAL) |
| MD04G1091200 | 2.157938 | 1.122646 | 1.215948 | xyloglucan endotransglucosylase/hydrolase protein 32 (XTH32) |
| MD15G1106700 | 2.713845 | 0.684101 | 1.049068 | photosystem II stability/assembly factor (HCF136) |
| MD04G1134900 | 2.699468 | 0.613069 | 1.479164 | tropinone reductase (TR) |
| MD04G1043200 | 1.791625 | 0.009874 | 1.283067 | retrovirus-related Pol polyprotein from transposon TNT 1-94 (KK1) |
| apple_newGene_599 | 1.817898 | 0.040539 | 1.791498 | isoaspartyl peptidase/L-asparaginase 3 (IYP3) |
| **Cluster 4** |  |  |  |  |
| MD02G1044000 | 0.146081 | 1.834904 | 1.138896 | thiamine pyrophosphokinase 1-like (TPS1-LIKE) |
| MD13G1164200 | 0.060144 | 1.711094 | 1.005118 | sucrose synthase 2-like (SUS2-LIKE) |
| MD17G1131600 | 0.183898 | 1.653238 | 0.874585 | wall-associated receptor kinase-like 8 (WAK8) |
| apple_newGene_1981 | 0 | 1.483559 | 1.004322 | BZIP domain class transcription factor (BZIP) |
| MD17G1140000 | 0.463013 | 1.675222 | 0.234072 | xyloglucan endotransglucosylase/hydrolase 6 (XTH6) |
| MD13G1037900 | 0.154968 | 1.072321 | 0.616708 | disease-resistance locus receptor-like protein kinase-like 2.4 (DRKL2.4) |
| MD13G1268900 | 0.109089 | 1.415714 | 0.169513 | xyloglucan endotransglucosylase/hydrolase protein 22-like (XTH22L) |
| MD06G1073900 | 0.175379 | 1.388312 | 0.67118 | 3-ketoacyl-CoA thiolase 2 (ACAA2) |
| MD02G1274100 | 0.046462 | 1.991246 | 1.547266 | alpha-xylosidase 2 (XYL2) |
| MD10G1250500 | 0.832306 | 1.776785 | 1.775666 | wall-associated receptor kinase 5-like (WAK5) |
| MD15G1188000 | 0.292096 | 2.107573 | 1.432547 | trihelix transcription factor (ASIL2-like) |
| MD02G1273500 | 0.314355 | 1.425613 | 1.647936 | rust resistance kinase Lr10-like (RRKL10) |
| apple_newGene_2182 | 0.039788 | 1.410486 | 1.411723 | isocitrate dehydrogenase (IDH) |
| MD09G1152600 | 0.441857 | 2.569516 | 0.352039 | threonine dehydratase biosynthetic (OMR1) |
| MD16G1267200 | 0.218069 | 2.525763 | 0.269435 | xyloglucan endotransglucosylase/hydrolase 8 (XTH8) |
| MD16G1231000 | 1.341913 | 1.80114 | 1.818283 | 7-deoxyloganetin glucosyltransferase-like (UGTL 4) |
| MD10G1216900 | 1.202314 | 1.639714 | 1.024993 | malate dehydrogenase-like (MDL) |
| apple_newGene_904 | 0.100278 | 0.694199 | 0.957737 | lon protease 2-like (IL2) |
| apple_newGene_1506 | 0.108321 | 0.875035 | 0.774732 | uncharacterized protein LOC108174989 |
| apple_newGene_1056 | 0.080103 | 0.61307 | 0.648662 | triacylglycerol lipase 1-like (TL1-LIKE) |
| MD02G1274000 | 0 | 0.469037 | 0.397424 | disease-resistance locus receptor-like protein kinase-like 2.1 (DRKL2.1) |
| MD17G1280400 | 0.031476 | 0.608943 | 0.445077 | galactinol synthase 1-like (GolS1-like) |
| MD04G1029600 | 0.019515 | 0.183364 | 0.600842 | pectinesterase-like (PECSL) |
| MD11G1034700 | 1.118251 | 1.439393 | 1.586564 | thiamine pyrophosphokinase 1-like (TPS1-LIKE) |
| MD05G1342500 | 0.135923 | 0.745498 | 0.162695 | beta-glucosidase 47-like (BGLU47-LIKE) |
| MD17G1143800 | 0.312628 | 0.196881 | 0.043402 | (R)-mandelonitrile lyase-like (RMDL) |
| MD04G1135300 | 0.398534 | 0.171618 | 0 | tropinone reductase (TR) |
| MD04G1160100 | 0.614043 | 0.553421 | 0.220232 | pyruvate decarboxylase 3 (PDC3) |
| apple_newGene_1664 | 0.375778 | 0.334899 | 0 | V-type proton ATPase subunit a1-like (ATP6VA1) |
| MD13G1264200 | 0 | 0.118523 | 0.040573 | (R)-mandelonitrile lyase 3-like (RMDL3) |
| MD17G1132000 | 0 | 0.213964 | 0.117881 | wall-associated receptor kinase-like 22 (WAK22) |
| MD15G1371800 | 0.062326 | 0.136548 | 0 | 7-deoxyloganetin glucosyltransferase-like (UGTL 1) |
| MD17G1125400 | 0.04059 | 0.159328 | 0 | 7-deoxyloganetin glucosyltransferase-like (UGTL 2) |
| MD06G1188600 | 0.225741 | 0 | 0.24821 | pectate lyase-like (PLL) |
| MD02G1266500 | 0.784658 | 0.777094 | 0.184964 | pectinesterase/pectinesterase inhibitor 47 (PME47) |
| apple_newGene_1074 | 1.396676 | 0.48722 | 0.702983 | presequence protease 1 (PREP1) |
| apple_newGene_269 | 1.283906 | 0.159007 | 0.460412 | uncharacterized protein LOC103426825 |
| MD03G1065100 | 1.507439 | 0.397133 | 0.686392 | pyruvate decarboxylase 1-like (PDC1-LIKE) |
| MD02G1281400 | 1.380772 | 0.438817 | 1.116173 | 2-oxoglutarate dehydrogenase, mitochondrial-like (OGDH) |
| MD06G1120600 | 1.479332 | 0.117715 | 0.741771 | mitochondrial protein AtMg00810-like |
| MD01G1106300 | 1.288372 | 0 | 0.491125 | uncharacterized LOC103958368 |
| MD08G1082700 | 1.288209 | 0 | 0.588946 | uncharacterized LOC103415991 |
| apple_newGene_1859 | 1.130139 | 0.150688 | 0.728958 | chorismate mutase 3 (CM3) |
| apple_newGene_2634 | 1.010685 | 0.346591 | 0.459233 | zinc finger protein 4-like (ZNF4) |
| apple_newGene_2026 | 0.947464 | 0 | 0.288461 | pyruvate decarboxylase 1 (PDC1) |
| MD01G1213200 | 0.893247 | 0.014441 | 0.255205 | uncharacterized LOC103943531 |
| apple_newGene_1469 | 1.009902 | 0 | 0.301235 | protein (MOR1) |
| MD07G1053200 | 0.891313 | 0.333075 | 0.260695 | pectinesterase/pectinesterase inhibitor 48 (PME48) |
| MD16G1081500 | 0.863185 | 0.376358 | 0.655004 | uncharacterized protein LOC103402950 |
| apple_newGene_1696 | 0.69612 | 0 | 0.162771 | histidine-containing phosphotransfer protein 2-like (HP2-LIKE) |
| apple_newGene_1333 | 0.56046 | 0.062491 | 0.195055 | uncharacterized protein LOC108173984 |
| MD17G1124400 | 0.662672 | 0.271589 | 0.372036 | 7-deoxyloganetin glucosyltransferase-like (UGTL 3) |
| MD14G1195200 | 0.463819 | 0.127473 | 0.301473 | pectate lyase-like (PEL) |
| MD10G1316200 | 1.694352 | 0.647177 | 0.533636 | beta-glucosidase 47-like (BGLU47-LIKE) |
| apple_newGene_2154 | 2.010267 | 0 | 0.575872 | 3-ketoacyl-CoA thiolase 2 (ACAA2) |
| **Cluster 5** |  |  |  |  |
| MD16G1265300 | 0.979475 | 1.705674 | 2.254835 | (R)-mandelonitrile lyase 1-like (RMDL1) |
| MD09G1147500 | 0.627512 | 0.984535 | 2.563593 | transcription factor (JUNGBRUNNEN 1-like) |
| MD17G1126300 | 0.505116 | 1.132436 | 2.629571 | 7-deoxyloganetin glucosyltransferase-like (UGTL) |
| MD11G1070800 | 0.675285 | 0.963397 | 2.879404 | asparagine synthetase [glutamine-hydrolyzing] 1 (ASNS1) |
| MD03G1066000 | 0.892226 | 1.330543 | 3.307728 | asparagine synthetase [glutamine-hydrolyzing]-like (ASNSL) |
