# Supplementary material for: Transcription profiles reveal sugar and hormone signaling pathways mediating tree branch architecture in apple (Malus domestica Borkh.) grafted on different rootstocks
Source: PLoS One. 2020 Jul 24;15(7):e0236530. doi: 10.1371/journal.pone.0236530 (PMC7380599; doi:10.1371/journal.pone.0236530)
Supplement: S5 Table — (DOCX) [file pone.0236530.s007.docx]

**Table S5 Information of selected phytohormone related genes in leaves of apple trees on different rootstocks.**

| **Gene ID** | **FPKM** | | | **Annotation** |
| --- | --- | --- | --- | --- |
|  | **VR** | **DIR** | **DSR** |  |
| **Cluster 1** |  |  |  |  |
| apple_newGene_1981 | 0 | 1.483559 | 1.004322 | BZIP domain class transcription factor (BZIP) |
| MD06G1010900 | 0.489355 | 1.791617 | 0.824299 | ABA 8'-hydroxylase 4 (ABA8OX4) |
| apple_newGene_158 | 0.000237133 | 0.62122 | 0.187183 | manganese superoxide dismutase 2 (Mn-SOD2) |
| apple_newGene_150 | 0 | 0.367606 | 0.166206 | SKP1-like protein 21 (SKPL21) |
| MD15G1082600 | 0.32539 | 1.126171 | 0.422705 | abscisic acid 8'-hydroxylase 4-like (ABA8OX4-LIKE) |
| apple_newGene_901 | 0.938473 | 0.060233 | 0.318745 | protein phosphatase 2C 27 (PP2C) |
| apple_newGene_1696 | 0.69612 | 0 | 0.162771 | histidine-containing phosphotransfer protein 2-like (HP2L) |
| **Cluster 2** |  |  |  |  |
| apple_newGene_2022 | 0 | 4.497999 | 4.355282 | RGL2-2, partial |
| MD03G1014200 | 3.151545 | 4.884741 | 5.510437 | peroxidase A2-like (PODA2L) |
| MD05G1081400 | 2.39091 | 3.41447 | 3.341143 | peptidyl-prolyl cis-trans isomerase (CYP40) |
| MD13G1185100 | 6.955576 | 7.137732 | 8.161838 | stem-specific protein (TSJT1) |
| MD12G1259500 | 2.814498 | 3.381881 | 4.813546 | auxin efflux carrier component 8 (PIN8) |
| MD04G1243800 | 0.123597 | 0.528054 | 2.113366 | auxin efflux carrier component 3 (PIN3) |
| apple_newGene_1867 | 2.68336 | 0 | 2.46893 | peptidyl-prolyl cis-trans isomerase (CYP19-3-like) |
| MD15G1268700 | 2.032005 | 0 | 1.046657 | NEDD8-conjugating enzyme Ubc12-like |
| apple_newGene_1992 | 2.56487 | 0 | 1.484367 | auxin response factor 7-like (ARF7L) |
