# Supplementary material for: Transcription profiles reveal sugar and hormone signaling pathways mediating tree branch architecture in apple (Malus domestica Borkh.) grafted on different rootstocks
Source: PLoS One. 2020 Jul 24;15(7):e0236530. doi: 10.1371/journal.pone.0236530 (PMC7380599; doi:10.1371/journal.pone.0236530)
Supplement: S6 Table — (DOCX) [file pone.0236530.s008.docx]

**Table S6 Information of selected transcription factors in leaves of apple trees on different rootstocks.**

| **Gene ID** | **FPKM** | | | **Annotation** |
| --- | --- | --- | --- | --- |
|  | **VR** | **DIR** | **DSR** |  |
| **Cluster 1** |  |  |  |  |
| MD02G1217900 | 3.650959815 | 0.852152 | 1.625871 | ethylene-responsive transcription factor (ERF034-like) |
| apple_newGene_426 | 3.562896501 | 0 | 1.752642 | E3 ubiquitin-protein ligase (MARCH8-like) |
| MD02G1234500 | 2.740752729 | 1.127044 | 2.185179 | pentatricopeptide repeat-containing protein (PPR) |
| apple_newGene_1867 | 2.683359599 | 0 | 2.46893 | peptidyl-prolyl cis-trans isomerase (CYP19-3-like) |
| apple_newGene_1992 | 2.564870203 | 0 | 1.484367 | auxin response factor 7-like (ARF7-LIKE) |
| MD02G1185900 | 1.949959431 | 0.15619 | 0.754175 | protein SHORT-ROOT (SHR) |
| MD10G1022700 | 2.339532163 | 0.838425 | 1.252374 | uncharacterized LOC103407656 |
| MD15G1106700 | 2.713844622 | 0.684101 | 1.049068 | photosystem II stability/assembly factor (HCF136) |
| apple_newGene_1981 | 0 | 1.483559 | 1.004322 | BZIP domain class transcription factor (BZIP) |
| MD11G1311300 | 0.26900533 | 1.052947 | 0.584166 | probable transmembrane GTPase FZO-like (FZL) |
| apple_newGene_3 | 0 | 0.21601 | 0.594124 | replication protein A 70 kDa DNA-binding subunit (RPA1) |
| apple_newGene_1056 | 0.080102761 | 0.61307 | 0.648662 | triacylglycerol lipase 1-like (TGL1L) |
| MD10G1306300 | 0.78529355 | 0 | 0.198826 | uncharacterized LOC103446611 |
| apple_newGene_2634 | 1.010684849 | 0.346591 | 0.459233 | zinc finger protein CONSTANS-LIKE 4-like (COL4) |
| **Cluster 2** |  |  |  |  |
| apple_newGene_1660 | 0.165334209 | 4.145667 | 3.650399 | uncharacterized protein LOC103958785 |
| MD10G1276200 | 1.056045201 | 3.63962 | 3.236315 | homeobox protein SHOOT MERISTEMLESS-like (STL) |
| apple_newGene_2022 | 0 | 4.497999 | 4.355282 | RGL2-2 |
| MD12G1072500 | 0.954817208 | 2.455626 | 2.096369 | GTP-binding protein TypA/BipA homolog (TYPA) |
| apple_newGene_1322 | 0.863410423 | 0.810045 | 2.7833 | triacylglycerol lipase 2-like (TGL2L) |
| MD15G1302900 | 2.977333844 | 3.470454 | 4.303028 | homeobox-leucine zipper protein (HAT22-like) |
| MD15G1384600 | 4.889784589 | 5.557639 | 6.688822 | MADS-box protein (SVP-like) |
| MD16G1084400 | 4.504380983 | 4.141198 | 5.248364 | two-component response regulator (ARR5) |
| MD07G1232800 | 6.44968517 | 7.397472 | 7.461928 | uncharacterized LOC103408239 |
| MD07G1013000 | 4.5572855 | 3.017218 | 4.121882 | uncharacterized LOC103945891 |
| apple_newGene_171 | 3.405946084 | 2.424607 | 2.699846 | ATP-dependent zinc metalloprotease (FtsH-like) |
| MD07G1248600 | 3.623924203 | 5.671081 | 4.360296 | ethylene-responsive transcription factor (ERF105) |
| MD06G1034300 | 4.373775796 | 6.458772 | 4.611009 | transcription factor (MYC2) |
| MD16G1274200 | 3.824940925 | 5.826109 | 3.698896 | transcription factor (MYC2-like) |
